# Hollow Filaments Synthesized by Dry-Jet Wet Spinning of Cellulose Nanofibrils: Structural Properties and Thermoregulation with Phase-Change Infills

**DOI:** 10.1021/acsapm.2c00177

**Published:** 2022-03-21

**Authors:** Guillermo Reyes, Rubina Ajdary, Maryam R. Yazdani, Orlando J. Rojas

**Affiliations:** † Biobased Colloids and Materials, Department of Bioproducts and Biosystems, School of Chemical Engineering, Aalto University, Espoo FI-00076, Finland; ‡ Department of Mechanical Engineering, School of Engineering, Aalto University, Espoo FI-02150, Finland; § Bioproducts Institute, Department of Chemical & Biological Engineering, Department of Chemistry and Department of Wood Science, The University of British Columbia, 2360 East Mall, Vancouver, BC V6T 1Z3, Canada

**Keywords:** nanocellulose, spinning, hollow filaments, phase-change materials, functional textiles, energy storage, wearables

## Abstract

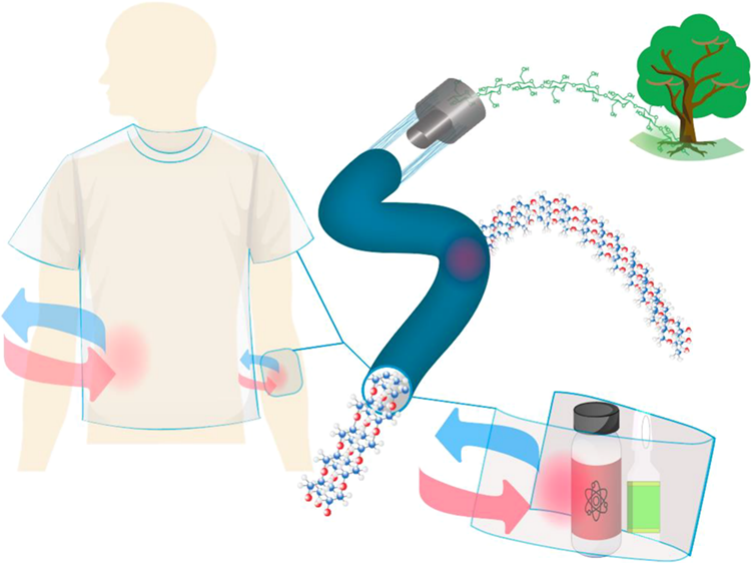

We use dry-jet wet spinning in a coaxial configuration by extruding an aqueous colloidal suspension of oxidized nanocellulose (hydrogel shell) combined with airflow in the core. The coagulation of the hydrogel in a water bath results in hollow filaments (HF) that are drawn continuously at relatively high rates. Small-angle and wide-angle X-ray scattering (SAXS/WAXS) reveals the orientation and order of the cellulose sheath, depending on the applied shear flow and drying method (free-drying and drying under tension). The obtained dry HF show Young’s modulus and tensile strength of up to 9 GPa and 66 MPa, respectively. Two types of phase-change materials (PCM), polyethylene glycol (PEG) and paraffin (PA), are used as infills to enable filaments for energy regulation. An increased strain (9%) is observed in the PCM-filled filaments (HF-PEG and HF-PA). The filaments display similar thermal behavior (dynamic scanning calorimetry) compared to the neat infill, PEG, or paraffin, reaching a maximum latent heat capacity of 170 J·g^–1^ (48–55 °C) and 169 J·g^–1^ (52–54 °C), respectively. Overall, this study demonstrates the facile and scalable production of two-component core-shell filaments that combine structural integrity, heat storage, and thermoregulation properties.

## Introduction

The use of renewable resources in advanced bioproducts is the subject of current interest,^1^ given the promise of positive environmental impacts.^2^ For instance, innovative applications are being developed in the area of biomedical devices, tissue engineering, cell scaffolds, and smart textiles.^3−5^ The markets associated with the latter industry segment benefit from traditional cellulose-based materials and their potential “*decarbonization*” benefits.^4,6^ Hence, related efforts consider biobased polymeric structures and cellulose nanofibers (CNF) as platforms for synthesizing strong, functional materials.^7,8^ For example, spinning has been suggested as a technology to achieve the maximum theoretical mechanical performance shown by individual cellulose building blocks (CNF) but in the form of textile filaments. Such strength optimization is best achieved in highly oriented cellulose fibrils under shear or flow-focusing effects.^9−12^

Cellulose oxidation by (2,2,6,6-Tetramethylpiperidin-1-yl)oxyl (TEMPO) has been shown as a regioselective (C6) process leading to anionic cellulose nanofibrils (TOCNF), which have been used to produce robust and optically transparent materials.^13^ These and other properties are benefited from an extensive degree of fibrillation,^14^ and simultaneous low-to-negligible cytotoxicity.^15^ Hence, TOCNF spinning has been used to produce filaments for fire retardants,^16^ wound dressing materials, cell cultivation scaffolds,^17^ fiber-reinforced composites,^10^ tissue engineering systems,^18^ conductive fibers,^19^ drug carriers,^20,21^ and composites with phase-change materials (PCM).^22^

Cellulose has been proposed to fabricate hollow fibers, mainly for the reparation of nerve injuries and in dialysis.^23−28^ Kell and Mahoney,^23^ patented a method that combined cellulose acetate with polyethylene glycol and glycerin to produce hollow filaments (HF) in artificial kidneys. Later, Terumo Corporation patented a technology based on cellulose cuprammonium and cellulose acetate to produce HF for artificial dialysis.^24−26^ Luo et al.^27^ and Zhao et al.^28^ have also proposed cellulose composites with soy protein to fabricate HF for implants in the reparation of rats’ sciatic nerves. Unfortunately, the few reports about cellulose-based HF offer little insights into their mechanical performance and wet stability.

Recently, nanocellulose-based hollow fibers were produced using a triaxial head extruder composed of an inner conductive polymer solution (PEDOT/PPS) layer. The latter work highlighted the prospects of producing short filaments in small batches. However, the continuous production of uniform HF remains challenging.^29^ A recent report indicates HF formed by cellulose nanofibrils that form laminated rectangular sheets,^30^ suitable for rolling around a mandrel (1.65 mm diameter) to produce two-layered HF. However, these HF exhibited poor mechanical stability because of delamination and unrolling. Hence, despite the promising properties of nanocellulose,^7,8^ no suitable method has been made available to continuously produce CNF HF with the mechanical strength expected for any use.

Some filament systems have been considered for their potential use in nerve surgery or reparation,^31^ advanced biomedical devices,^32^ controlled release of growth factors, or targeted therapeutics.^32^ For related uses and compared to unmodified CNF, TEMPO-CNF has shown to produce suitable mechanical properties and light transmittance.^33−36^ Clearly, an opportunity emerges in the area of smart textiles and wound healing if cellulose-based filaments are combined with PCM. However, to our knowledge, the use of HF as carriers for PCMs has not been attempted so far. Thus, we introduce a simple method to stabilize a continuous air gap within a nanocellulose shell, yielding stable and self-standing nanocellulose-based hollow filaments (HF). We further demonstrate the infilling of PCMs using the hollow filaments for uses in thermal energy management, with a potentially significant impact on wearable devices, wound dressing materials, and protecting temperature-sensitive goods.^37,38^ For instance, the system can be further considered for bandages and wound/burn dressings, given the liquid transport and antibacterial features that can be added,^39−41^ while maintaining the temperature in contact with the skin^42^ or in biomedical patches and implantable energy-storage devices.^43,44^ The cellulose nanofiber shell component brings additional benefits of cost-effectiveness, biocompatibility, lightweightness, safety, and sustainability.^42,45^

## Materials and Methods

### Dry-Jet Wet Spinning

Fines-free Kraft bleached birch fibers were supplied by UPM Finland (lignin % 0.18, DP 4700) and used to prepare hydrogels of TEMPO-oxidized cellulose nanofibrils (TOCNF) (2% w/w content). TOCNF was produced according to a previously reported procedure,^46,47^ using 2,2,6,6-tetramethylpiperidine-1-oxyl or TEMPO (CAS No. 2564-83-2, purity >98%), sodium bromide NaBr (CAS No. 7647-15-6, purity >99%), sodium hypochlorite NaClO (CAS No. 7681-52-9, reagent grade), and sodium hydroxide (NaOH) (CAS No. 1310-73-2, purity >99%), all acquired from Sigma-Aldrich. For each gram of dry fibers, 0.13 mmol TEMPO and 4.65 mmol NaBr were dispersed in deionized water. The oxidized fibers were homogenized in a microfluidizer (1 pass at 2000 bar, Microfluidics M-110P, International Corporation, USA). The produced TOCNF hydrogel was characterized for the carboxylic group content by conductometric titration according to standard SCAN-CM 65:0278,^11^ which indicated a total charge of 1.30 (0.05) mmol_COOH_/g_fiber_. The TOCNF morphology was analyzed using an atomic force microscopy (AFM) (Digital Instruments Multimode Atomic Force Microscope, Bruker, U.K.) instrument, following a previously reported procedure.^7,48^ The fibrils had an average width of 25(7) nm with an aspect ratio of L/D > 100.

The obtained TOCNF hydrogels were de-aired in a planetary centrifugal mixer (THINKY AR-250, JAPAN) and transferred to a syringe (Henke Sass Wolf, 60 mL, Luer lock, soft jet ) and stored at 5 °C overnight. The TOCNF-C hydrogels (2% w/w) were extruded using a syringe pump (CHEMYX, Model FUSION 6000, USA) equipped with a coaxial spinning nozzle (Ramé-Hart Instrument CO), using a Gauge 13 for the outer needle with an inner diameter Φ_e_ = 1.8 mm. Three inner syringes were used Gauge 21, 19, and 17 corresponding to small, medium, and larger outer diameters, Φ_i_ of 0.813, 1.07, and 1.47 mm, respectively. Coagulation in a bath produced HF and, depending on the geometry of the coaxial system, yielded the respective sizes, namely, small, medium, and large HF (HF-s, HF-m, HF-l, respectively). Room temperature (20 °C) was used during spinning, and the coagulation was conducted in an acid bath 0.01 M HCl (HCl ACS reagent, 37%, and Milli Q type I water). The acid bath conditions were selected according to previous experience,^10,49,50^ which indicated instantaneous coagulation of TOCNF in contact with water at pH = 2 (0.001 M HCl), where protonation of carboxylate groups occurs, reducing the electrostatic repulsion according to Derjaguin–Landau–Verwey–Overbeek theory (DLVO).^50^ This condition leads to the diffusion of water from the extruded nanocellulose hydrogel, drawing the fibrils together and leading to their solidification as a filament.

The TOCNF was extruded at a volumetric rate *Q* = 4 mL·min^–1^, which was optimized for stable, defect-free formation as HF. Lower TOCNF extrusion rates <4 mL·min^–1^ formed very soft HF (breaking when contacting the regeneration bath). High rates produced filaments that were not amenable for pickup. The shear rates (γ) during the dry-jet wet spinning was calculated by assuming plug flow conditions, eq 1:^9^
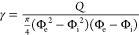
1

Given the different coaxial needles used, the shear rate depended on the inner diameter: γ = 33, 56, and 238 s^–1^ for the small, medium, and large diameter Φ_i_. It should be noted that the larger inner diameter Φ_i_ translates into a smaller annular area and hence a higher shear rate. The HF were extruded using a vertical air gap (2 cm), following a previously reported setup^11^ and noting that larger gaps led to the collapse of the regenerated HF.

**Figure 1:**
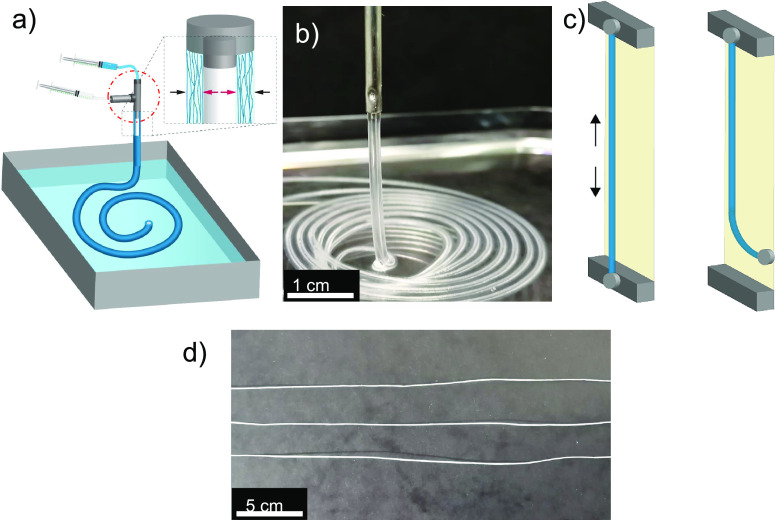
HF spinning: (a) TOCNF hydrogel and air extrusion in a coaxial setup. (b) Photograph of a long hollow filament formed from the extruded TOCNF hydrogel on the coagulation bath. (c) Schematic illustration of the drying process used for small, medium (maximum tension), as well as large size (moderate tension) hollow filaments. (d) Photograph of dry hollow filaments.

Air was continuously pumped through the inner needle (1 mL·min^–1^) and prevented wall collapse for HF formation (the leading end of the HF was sealed with tweezers). After optimization, the air and the TOCNF flow pump rates were selected to produce stable HF. It should be noted that no drawing was applied, and the filaments were left to accumulate in the coagulation bath for as long as suitable, reaching several meters in length (see Figure 1). The coagulation time can be arbitrarily taken as the residence time (the time the hydrogel system is immersed in the coagulation bath). However, the actual coagulation process can be swift. Given the long holding time, this latter condition is applied in our work. After holding in the acidic bath, the HF were collected and cut into small pieces (0.5 m), fixed on their ends, and dried for 24 h at room temperature. The HF produced with inner needles gauge 21 and 19 (small and medium core diameters, respectively) withstood the drying tension. The larger ones (inner needle gauge 17) fractured during drying (because of their thinner wall); therefore, these samples were dried under no tension, leading to a 2/3 shrinkage relative to their initial length. It is worth noting that the HF were produced following a semibatch operation. However, continuous hollow filament extrusion was successfully tested, indicating the possibility for scalability (see video supplied as the Supporting Information; Spinning process.mp4).

### HF and HF-PCM System

Two different PCMs (polyethylene glycol 4000, PEG, and paraffin, PA) were used to fill the dry HF by means of capillary effects. For this purpose, the HFs were placed in a beaker containing the respective PCM (70 °C, 200 mbar vacuum pressure). The melted PCM filled the hollow filament, and any excess PCM remaining on the outer surface was removed. The diameter and surface morphology of HF and HF-PCM were accessed using scanning electron microscopy (SEM) (ZEISS SIGMA VP, Germany). Before imaging, the samples were vacuum-dried for 18 h overnight and subsequently sputtered with Au/Pt (∼7 nm, Emitech K100X). The images were analyzed using ImageJ.^51^ Swelling was assessed using the SEM (dry) and wet diameters. The samples’ wet mechanical and morphological features were assessed after immersion of the filaments in distilled water overnight. The filament dimensions in the wet state were obtained using an optical microscope (Leica DM 750 Microsystems, Germany, camera ICC50HD). For this purpose, the samples were placed on a glass slide, and lighting was adjusted using an external source (Lampe Fiber Optic Fi. L-100). The swelling ratio was determined according to eq 2:
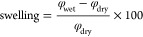
2where φ_dry_ and φ_wet_ correspond to the dry and wet diameters, respectively, HF-PCM infill morphologies were also observed under SEM.

### Rheological, Specific Surface Area, and Strength Properties

The shear rheology of TOCNF gels was studied under steady and oscillatory modes using an Anton Paar Physica MCR 302 (Anton Paar GmbH, Austria). The rheometer was equipped with a Peltier hood H-PTD 200 for controlled temperature and humidity. All measurements were performed at 25 °C, and the tests were carried out with a parallel plate geometry (25 mm diameter and 1 mm gap). Nitrogen sorption measurements were performed at 77 K on Micromeritics ASAP 2020 and TriStar II 3020 instruments. The surface area was calculated according to the Brunauer–Emmett–Teller (BET) theory. According to the Barrett–Joyner–Halenda (BJH) theory, the pore size distribution was applied to the adsorption and desorption branches of the BET isotherm. The samples were degassed under a vacuum at 60 °C overnight. The filament mechanical properties were studied using a Universal Tensile Tester Instron 4204, 100 N load cell, and test speed 1 mm·min^–1^. The samples were analyzed according to the ASTM D3822/D3822M standard. Ten replicas of each sample were taken for the mechanical tests.

### Small and Wide-Angle X-Ray Scattering

The orientation, crystallinity index, and lateral crystallite size (200) of cellulose in the HF and HF-PCM were obtained using a bench beamline equipment small-angle and wide-angle X-ray scattering (SAXS/WASX) device (Xenocs, Xeuss 3.0, U.K.). The generator worked at 45 kV and 200 mA, with Cu Kα radiation. Background correction due to the sample holder and the air was made by subtracting the sample diffractogram data with the corresponding blank data (without sample). Every sample was measured in three different positions. The deconvolution procedure (Gaussian functions) was performed using fityk.^52^ The Herman’s orientation parameter was calculated by the integration of the azimuthal intensities according to eq 3:^53^
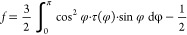
3where φ is the azimuthal angle, and τ(φ) represents the normalized azimuthal intensity distribution after subtracting the isotropic contribution. The lateral crystallite size, τ(Å) was calculated using the Scherrer equation,^54^and the reference crystallinity index (CI) was calculated using the Segal method.^11,55^

### Water and Oil Flow and Leakage Tests

Two tests were performed at pump rates of 1 mL·min^–1^ (see videos provided as the Supporting Information; Conduit oil transport.mp4 and conduit water transport.mp4). Dyed water (methylene blue) and vegetable olive oil were used to observe liquid flow/transport inside the conduit. The HF (small, medium, and large diameter) were filled with the PCMs (PEG 4000 and Paraffin 52–54 °C) and sealed on the ends with Loctite super glue and further tested for leakage in an oven at 90 °C for 2 h. The test was repeated three times for each sample. As a reference, an unsealed sample was placed in every test (see the Supporting Information).

### Thermal Properties

Differential scanning calorimetry (DSC) was conducted on a Netzsch DSC204F1 Phoenix instrument and a DSC instrument (TA Instruments, MT-DSC Q2000). The DSC samples consisted of HF (10–15 mg) placed in a standard aluminum crucible sealed with a lid. The sample was exposed to a dynamic DSC program covering the 0–80 °C temperature range. The reproducibility of the phase-change storage was followed using 100 consecutive heating–cooling cycles performed at a 5 K·min^–1^ scan rate. The specific heat capacity (*C*_p_) was determined using the Sapphire correction *C*_p_ method.

## Results and Discussion

### Hollow Filaments

A stable gel-type behavior for TOCNF dope rheology was observed with an elastic modulus one order of magnitude above the loss modulus (Figure S1). In addition to the solid-like behavior (*G*′ ≫ *G*″), we observe shear thinning at high shear rates and CNF alignment above the shear rate of 10 s^–1^; above this point, the dope exhibited the birefringence characteristic of well-aligned TOCNF underflow.^56^ (Figure S1).

As reported earlier,^9,56^ the fibril alignment is favored by shear forces and can be improved in the presence of an air gap before contact with the coagulation bath.^57^ Herein, the dopes were extruded using a vertical air gap of 2 cm with a shear rated well above 10 s^–1^; such shear rates facilitated the TOCNF alignment and promoted a drop in the zero-shear viscosity by more than two orders of magnitude. Additionally, the airflow in the core stabilized the extruded HF by balancing the external forces (atmospheric pressure and possible stress during coagulation, see black and red arrows in Figure 1a).

**Figure 2:**
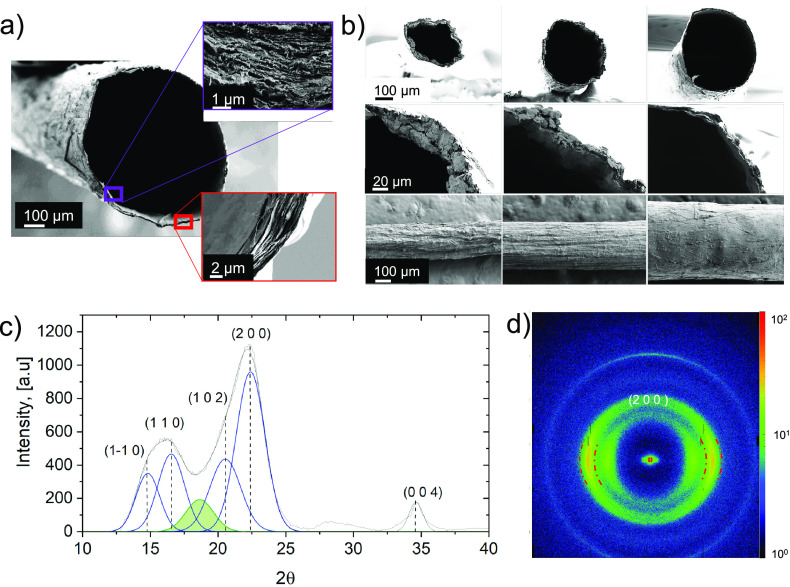
HF morphology and crystal structure: (a) cross-section area of the large-diameter hollow filament, HF-l. (b) Different size HF (HF-s, HF-m, HF-l, from left to right), cross-section (first row) and wall thickness (second row), and surface morphology (third row). (c) X-ray diffraction pattern of HF-s. (d) Azimuthal intensity of X-ray diffraction peaks of HF-s placed orthogonal to the beam (red, dash lines indicate the area of maximum intensity).

The airflow (1 mL·min^–1^ continuous pump, Figure 1a) created an internal pressure that prevented the collapse of the cylindrical walls during extrusion and coagulation. The internal pressure created can be calculated considering the ideal gas law for air (eq 4):
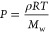
4where *T* is the temperature, 20 °C, and the density (ρ) and molecular weight (*M*_w_) of air correspond to 1.204 kg·m^–3^and 28.97 g·mol^–1^, respectively.^58^ The calculated inner pressure was *P* = 103,020 Pa, a pressure that was slightly higher than the atmospheric value (*P* = 101 kPa); this pressure was sufficient to maintain the stability on the extruded dope and to preserve a continuous air gap inside the filaments (Figure 1b, see also the Supporting Information video). After cutting, washing, and drying, 0.5 m long HF were dried under tension (Figure 1c), yielding three types of HFs according to the diameters (Figure 1d). The morphology and dimension of the HF are included in Figure 2.

Figure 2a shows the cross-section of HF-l (φ = 670 μm and Δφ = 14 (3) μm wall) that consists of well-organized concentrical layering (see red and violet squares in Figure 2a), following the expected pattern of a laminar flow.^59^Figure 2b displays the morphologies of the three different produced: small (HF-s), medium(HF-m), and large (HF-l), from the left to right, respectively. The smaller HF-s show more wrinkling than larger sizes because of the thick wall (left and right columns in Figure 2b). It should be noted that smaller and medium HF were dried under tension, but the large-diameter HF were dried under moderate tension (Figure 1c). The surface morphology results from the combined effect of wall thickness and drying tension, potentially impacting the TOCNF crystal structure and HF mechanical performance, as discussed in the following sections.

### HF Structural Characteristics

The nanofibril orientation in the HF wall was assessed from WAXS. Figure 2c shows a typical X-ray diffraction pattern for one sample (no significant differences were observed for the different HF sizes, see Figure S2 and Table S1). The HF presented the typical crystal structure of cellulose Iβ,^60^ with an average crystal lateral size of 35 Å and a CI of 75%. The peaks azimuthal distribution intensities were used to compute Herman’s orientation parameter, and no significant differences were observed in the crystalline structure for the different HFs (Tables S2 and S3). Tables S2 and S3 present the morphologies, surface area, porosities, and Herman’s orientation parameters for the HFs. As previously discussed, the HFs presented contrasting surface morphologies and wall thicknesses (Figure 2b). As a result, minor differences in the specific surface area were found. The HF-s presented the highest surface area (6.2 m^2^·g^–1^) and an average Herman’s orientation parameter 38% higher than the large-diameter HF-l. The medium-sized HF-m presented an orientation parameter only 23% higher than HF-l. The alignment gained upon extrusion for HF-l was lost during drying under moderate tension, which influenced the mechanical performance, Table S4. The densification and alignment in HF-s positively impacted the mechanical performance, Table S4; these filaments reached a value of up to 9 GPa for the Young modulus, 66 MPa tensile strength, and 2.1 MJ·m^–3^ toughness. These values are superior to the recent results for HF produced by rolling flat membranes of CNF (Young Modulus of 5.3 GPa).^30^ In comparison to other studies, our HFs performed better than solid TOCNF filaments produced under relatively similar conditions;^9,11,49,50,56^ however, our HF system did not reach the standard of technical textiles such as viscose,^61^ partially explained by the poor toughness of TOCNF fibers.^49^ Nevertheless, the mechanical strength is suitable or surpasses the needs of biomedical textiles and adds properties such as biocompatibility, lightweightness, safety, environmental impact, temperature regulation, and availability.^42,45^ Other considerations should be studied; for instance, some biomedical textiles are designed to lose mechanical strength (for instance, by more than 90%, following 30 days after implantation),^62^ for example, in sutures and wound-healing implants.

### HF-PCM Morphological and Structural Characteristics

**Figure 3:**
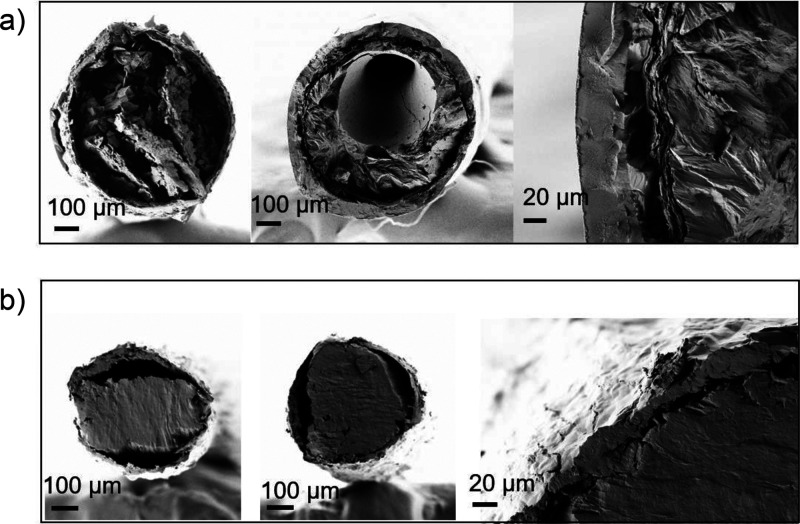
Morphology of HF filled with PCM (HF-PCM): (a) cross-section of HF-l-PEG and (b) HF-l-PA.

The HFs can continuously transport aqueous and oil phases (see the Supporting Information). For this purpose, HF-s and HF-l were selected as carriers for PEG and paraffin (our selected PCM). The HF filling was carried out at 70 °C in a vacuum oven (200 mbar) under the effect of capillary forces; for example, see HF-l filled with PEG (HF-l-PEG, Figure 3a) and paraffin (HF-l-PA, Figure 3b).

Figure 3a shows the cross-section of HF-l-PEG with the crystallized PEG (Figure 3a, left side). It indicates the PEG flow along the filament via wetting and hydrophilic interactions (Figure 3a, central image) with apparent adhesion to the HF walls (Figure 3a, right side). No leakage of HF-PEG (Figure S3a) was observed for all filaments following 2 h holding at 90 °C. The poor compatibility between paraffin and the HF is reproduced in Figure 3b (HF-l-PA), but as was the case of PEG, no leakage was observed in HF-PA (Figure S3b). Moreover, the water and oil flow tests (see the Supporting Information) showed that the HF contained/transported (low to high viscosity) fluids with no signs of leakage. The results confirm that TOCNF-based HFs are effective PCM supports. Other characteristics such as the crystal structure, mechanical properties, and thermal properties were examined.

The X-ray diffraction peaks of the confined PCM (after subtracting the cellulose background signal) exhibited the same peaks as those observed for the reference materials (pure melted crystallized powder PCM), Figure S4. PEG-filled filaments included two peaks at *q*_1_ = 1.33 Å or (2θ = 19.2°), and *q*_2_ = 1.62 Å or (2θ = 22.9°), attributed to the typical PEG 4000 crystal structure;^63,64^Figure S4a. The peaks in HF-s-PEG were of higher intensity than HF-l-PEG and the reference material. The peak at *q*_1_ = 1.33 Å indicated Herman’s parameter of *P*_2_ = 0.18, compared to those of the large filament, *P*_2_ = −0.02, and the reference material, *P*_2_ = 0.01. The enhancement of PEG 4000 *q*_1_ plane orientation can be explained by the formation of a well-defined crystal structure in a narrower confined cylindrical space; however, no similar trend was observed for *q*_2_ (see Figure S5). In contrast, the paraffin-filled filaments (HF-PA) presented a typical SAXS/WAXS crystal structure of paraffin with melting points around 52–62 °C (Figure S4c).^65−68^ The first two prominent WAXS peaks (2θ >5° region) correlate with the principal planes (110) and (200) appearing around 2θ = 21°, and 2θ = 23° for crystalline microparaffin (melting point of ca. 53 °C).^65^ In the SAXS region (2θ < 5°), it is possible to detect the larger-scale crystal planes formed upon paraffin crystallization;^66−68^ also, it showed the planes located at 2θ = 2.4, 4.9, and 7.4° attributed to orthorhombic paraffin structures (*n-pentacosane*, m.p = 52–54 °C^66,67^ or *n-octacosane*, m.p = 57–62 °C^68^). It should be noted that paraffins are long-chain saturated alkane mixtures with the general formula *C_n_H*_2*n*+2_. The X-ray diffraction pattern of HF-PA was not different compared to that of the neat material. The distribution of the peak azimuthal intensity indicated no preferential orientation (Figure S4d). However, the results suggest a better-crystalized structure in the narrow cylindrical space during PCM flow. The crystal structure of HF-PCM, and the interfacial interaction between the materials, are expected to influence the mechanical strength of the filaments, as discussed next.

### Mechanical Performance of HF, HF-PEG, and HF-PA

**Figure 4:**
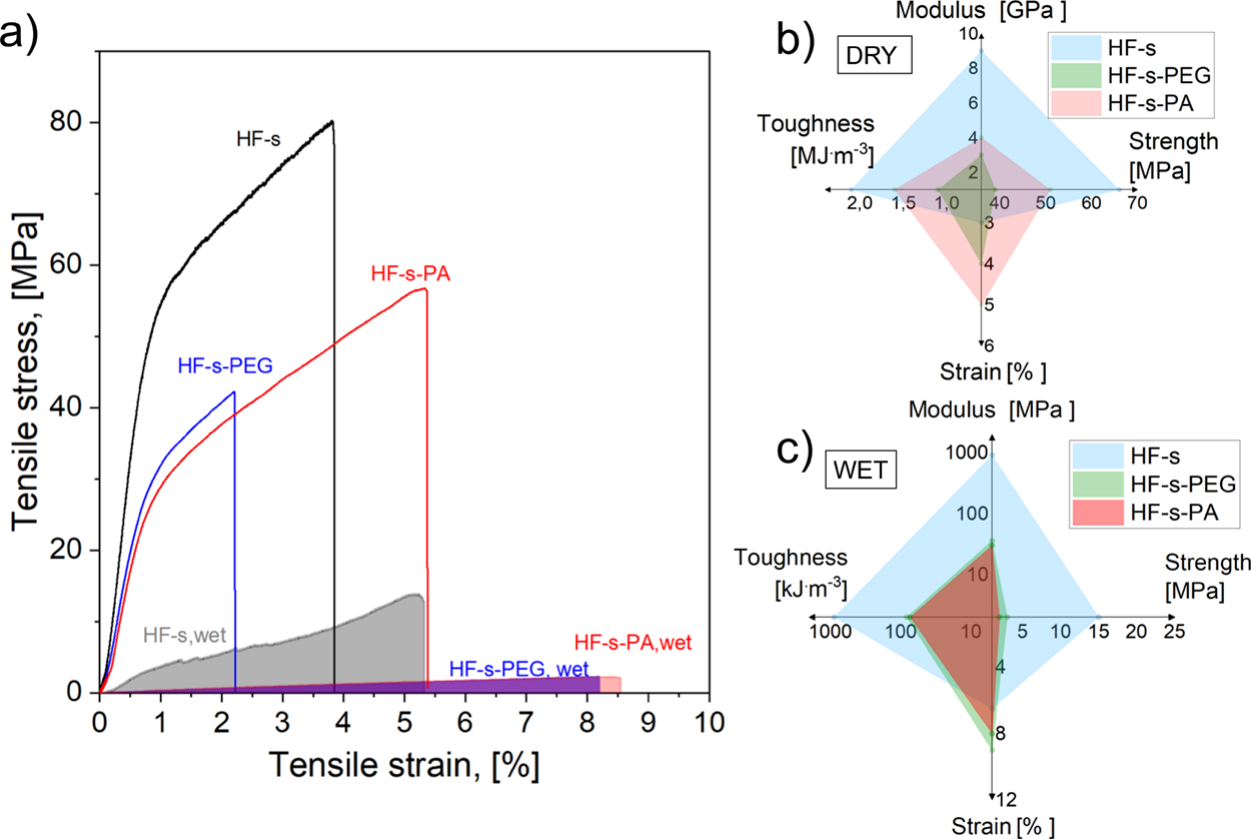
HF-s-PCM’s mechanical performance: (a) typical tensile profiles, (b) dry average mechanical properties, and (c) average wet mechanical properties.

The results of mechanical tests of HF-s-PCM in dry and wet states are shown in Figure 4. HF-s performed better than the PCM-filled filaments and compared favorably with respect to the recently reported hollow filaments produced by rolling flat membranes.^30^ The PCM-filled HFs were more stretchable in the wet state for both infilled PCMs and in the dry state only for PA (Figure 4a–c).

The paraffin-filled filaments showed better performance than the PEG-filled counterparts. PEG-HF hydrophilicity could potentially cause a detrimental effect on the filament performance because the filaments were conditioned in a 50% relative humidity room; in contrast, PA hydrophobicity led to better dissipation of elongational stress. However, the stretchability was improved by about 20% compared to the unfilled HF (Tables S5 and S6). Overall, the addition of the PCM did not enhance the mechanical performance, but as will be shown next, the thermal properties of the PCM confined in HF were largely maintained.

### HF-PCM Thermal Regulation

**Figure 5:**
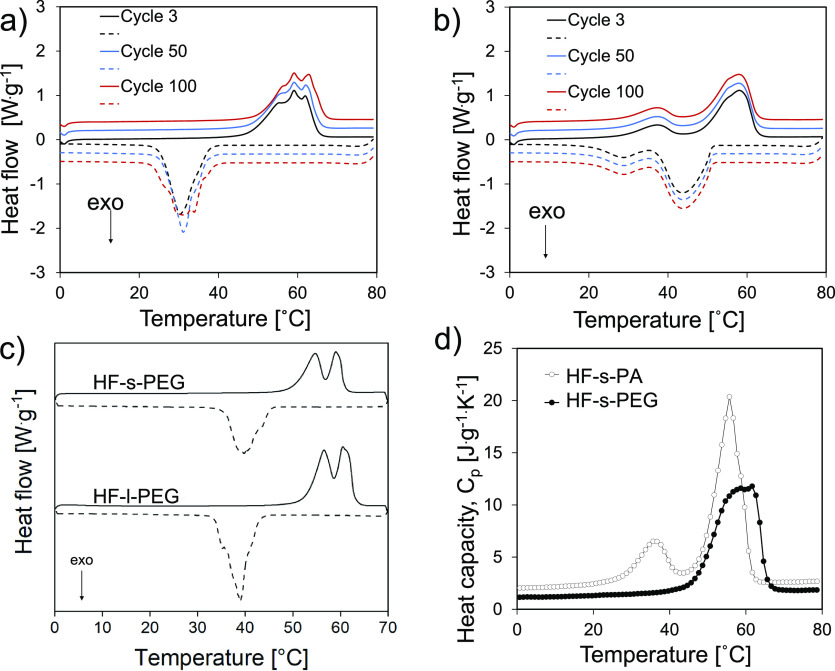
HF-PCM enthalpy and heat capacities profiles measured with a DSC running at 5 K·min^–1^: (a) HF-s-PEG, solid lines (heating), dashed lines (cooling). (b) HF-s-PA, solid lines (heating), dashed lines (cooling). (c) Size effect on DSC profiles (cycle 3) for HF-s-PEG and HF-l-PEG. (d) Specific heat capacities for HF-s-PA and HF-s-PEG.

PCMs usually have large heat for phase change and display sharp melting points;^44^ they also effectively store and release energy during phase transition by the evolution of sensible and latent heat. High heat capacities and latent enthalpies (*C*_pα_, *C*_pβ_, and Δ*H*^αβ^*)* are desirable for an efficient enthalpic process, eq S2.^69^ The DSC profiles for HF-PCM and the corresponding latent and specific heat capacities are shown in Figure 5. Figure 5a,b presents the cyclic DSC profiles for PEG and paraffin, respectively; the latent heat rate storage of the HF-PCMs, including the phase-change enthalpies and temperatures, is given in Table 1 (values for different cycles are given in Table S7).

**Table 1:** HF-PCM’s DSC Thermal Properties Following Three Heating/Cooling Cycles

filament	*T*_m_ [°C]	ΔHF [J g^–1^]	*T*_c_ [°C]	Δ*H*_c_ [J g^–1^]	PCM loading^a^ (%)
HF-s-PEG	58.8	137.2	32.2	–130.7	75
HF-l-PEG	60.2	170.1	38.9	–166.9	93
HF-s-PA	58.2	156.6	43.6	–155.5	74
HF-l-PA	54.4	169.4	49.45	–165.9	80

a PCM loading was calculated with reference to the reported latent heat for PEG4000 (182 J·g^–1^) and paraffin 52–54 °C (212 J·g^–1^), respectively.^22,70^

HF-s-PEG absorbed a latent melting heat of 137.2 J·g^–1^ with no considerable change after 100 consecutive cycles, Figure 5a. The corresponding heat release during crystallization was −130.7 J·g^–1^, which remained above −131 J·g^–1^ after 100 cycles (Table S7). Neat PEG shows 182 J·g^–1^ phase-change enthalpy,^22^ and 75% of PEG was successfully loaded in HF-s and 93% in HF-l, respectively.

For the small filled hollow filament, the stored heat through melting was 156.6 J·g^–1^, which remained above 152 J·g^–1^ after 100 consecutive cycles (Table S7). Likewise, the released heat corresponding to crystallization was −155.5 J·g^–1^, which remained above −151 J·g^–1^ after 100 cycles. Given that neat paraffin exhibits a phase-change enthalpy of 212 J·g^–1^,^70^ 74 and 80% paraffin were held in small- and large-diameter filaments, respectively. A highly repeatable thermal storage behavior is shown for all the HF-PCM, with the large diameter HF showing a thermal capacity close to that of the neat PCM (Figure 5c). It should be noted that a drop in more than 50% latent heat capacity compared to neat PCM is typical.^63^ PCM storage energy considers the combination of latent heat (Δ*H*) and sensible heat, determined by the specific heat capacity and temperature change (*C*_p_·Δ*T*), as shown in eq S2. The specific heat capacity of the liquid state is typically higher than that of the solid state,^69^ which is also the trend observed for the HF-s-PCM (Figure 5d). The specific heat capacities for the smaller filaments were extracted from Figure 5d at 20 °C for the solid-state and at 65 °C for the liquid state, respectively. The paraffin filaments showed higher *C*_p_ values of 2.59 and 2.47 J·g·K^–1^ for the liquid and solid states, respectively. These values are relatively smaller than those of PEG filaments, 1.87, and 1.32 J·g·K^–1^, respectively. Similar *C*_p_ values were previously reported for PCM-CNF composites.^22,63,70^ In summary, the result indicates the excellent prospects for HF produced from fully biobased, biocompatible cellulose nanofibrils.

## Conclusions

TOCNF was successfully spun into HF used as carriers of PCMs. The Young modulus of the HF reached values as high as 9 GPa, 170% higher compared to hollow filaments produced from flat membranes.^30^ The HFs did not exhibit delamination or instability and were shown to effectively hold PCMs of hydrophilic or hydrophobic nature (PEG 4000 and paraffin 52–54 °C). The filled hollow filaments, HF-s-PEG and HF-s-PA, exhibited a Young modulus of 3 and 4 GPa, and mechanical strength of 39 and 51 MPa, respectively. The produced filaments maintained the thermal exchange of the PCMs tested (up to 93% PCM loading or 170 J·g^–1^ latent heat of fusion), making the system suitable for associated applications such as smart textiles, wound dressing, and biomedical devices.

## Supporting Information Available

TOCNF SAXS/WAXS raw data, HF-PCM’s mechanical properties in dry and wet states, thermal properties after cycling, and videos to illustrate filament spinning and liquid transport (PDF)
Spinning process (MP4)
Conduit oil transport (MP4)
Conduit water transport (MP4)
