# Supplementary material for: Hollow Filaments Synthesized by Dry-Jet Wet Spinning of Cellulose Nanofibrils: Structural Properties and Thermoregulation with Phase-Change Infills
Source: ACS Appl Polym Mater. 2022 Mar 21;4(4):2908–16. doi: 10.1021/acsapm.2c00177 (PMC9003243; doi:10.1021/acsapm.2c00177)
Supplement: Supplementary file 1 — ap2c00177_si_001.pdf [file ap2c00177_si_001.pdf]

SUPPORTING INFORMATION

Hollow filaments synthesized by dry-jet wet spinning of cellulose nanofibrils: Structural properties and thermoregulation with phase change infills

Guillermo Reyes[*,ʃ], *Rubina Ajdary*[ʃ,ʒ], *Maryam R. Yazdani*[þ] *and Orlando J. Rojas*[*,ʃ,ʒ]

[ʃ]Biobased Colloids and Materials, Department of Bioproducts and Biosystems, School of Chemical Engineering, Aalto University, FI-00076, Espoo, Finland

[þ] Department of Mechanical Engineering, School of Engineering, Aalto University, FI-02150, Espoo, Finland

[ʒ] Bioproducts Institute, Department of Chemical & Biological Engineering, Department of Chemistry and Department of Wood Science, 2360 East Mall, The University of British Columbia, Vancouver, BC V6T 1Z3, Canada

*Corresponding authors: Guillermo Reyes, Email: guillermo.reyes@aalto.fi; Orlando J. Rojas, Email: orlando.rojas@ubc.ca

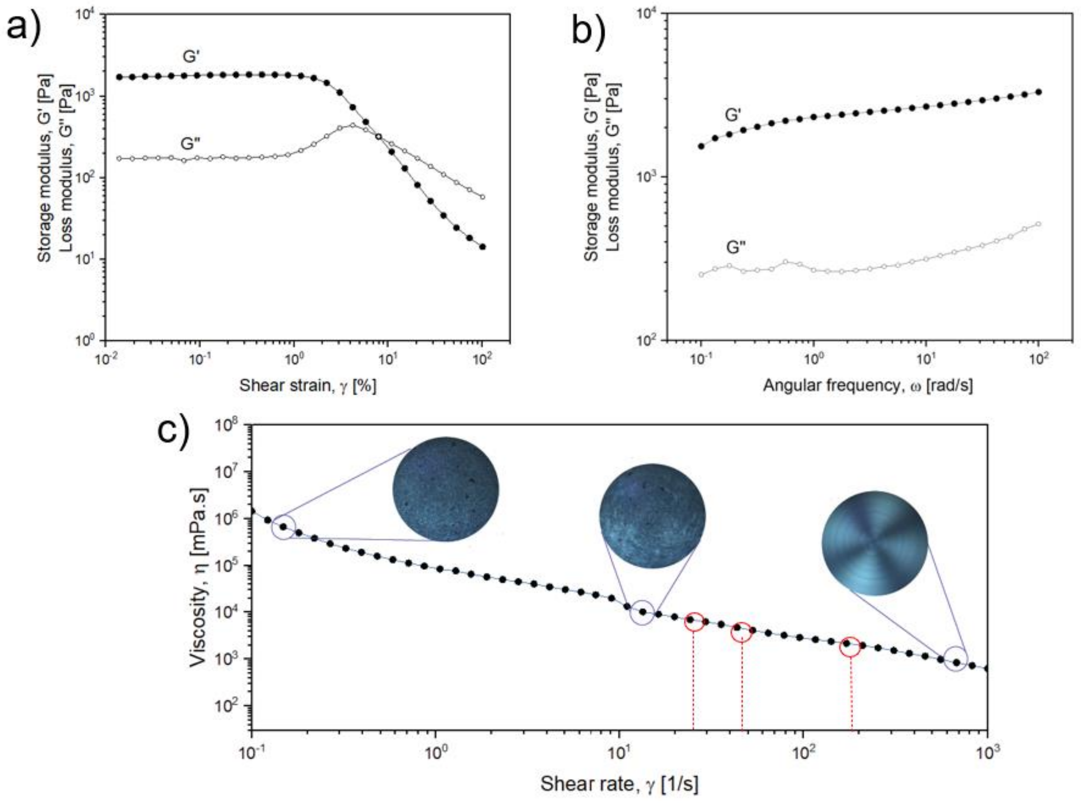

Figure S1. Rheology properties of TOCNF dope at 25 ºC: a) Linear viscoelastic region (LVR) at a constant angular frequency (1 rad·sec^{-1}). b) frequency swept at constant amplitude (0.1 %). c) dynamic viscosity with cross-polarized light camera snapshots (red circles show the experimental conditions to produce HF of small, medium, and large diameters, respectively).

TOCNF dopes (2 % w/w) were spun under optimal conditions under high shear rates, as shown in Figure S1. **Figure S1a** displays the linear viscoelastic region (LVR) of the TOCNF dope. The upper limit for the linear viscoelastic response was found below 1 % shear strain; additionally, the cross-over point was observed around 7 % shear strain (**Figure S1a**). Considering the LVR information, a frequency sweep at constant amplitude (shear strain 0.1 %) was performed to study the elastic (G') and loss modulus (G"), respectively (**Figure S1b**). Furthermore, the TOCNF underwent a disordered structure (see left side snapshot in Figure S1c) at low shear rates, passing through a shifting shear rate where the nanocellulose supramolecular structure started to align in the same direction of the deformation.

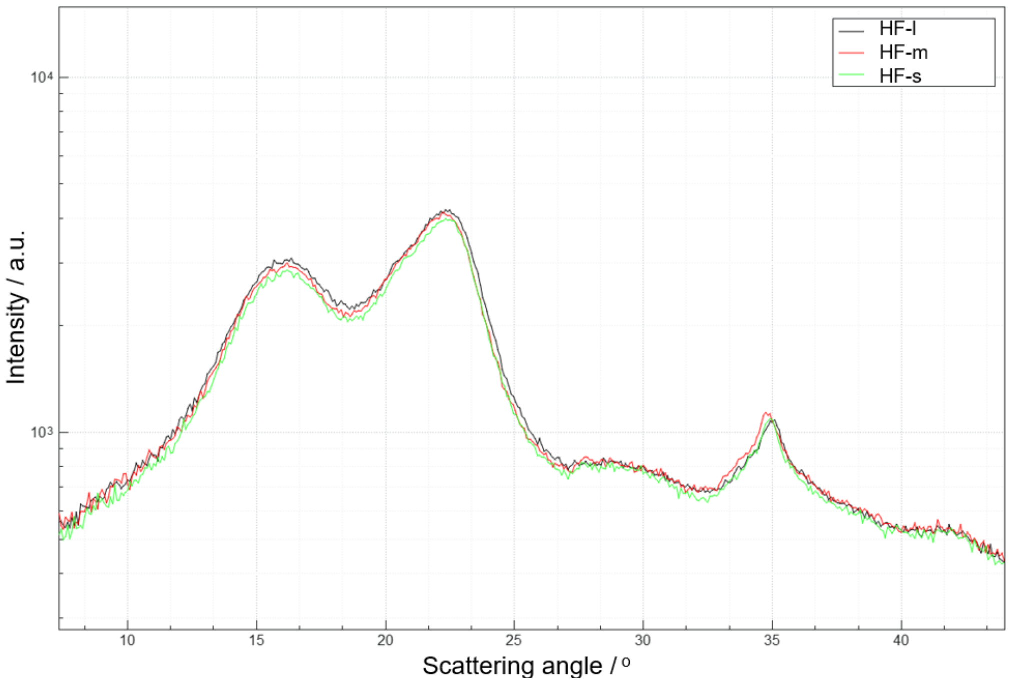

Figure S2. Raw data on X-rays diffraction peaks for large, medium, and small diameter

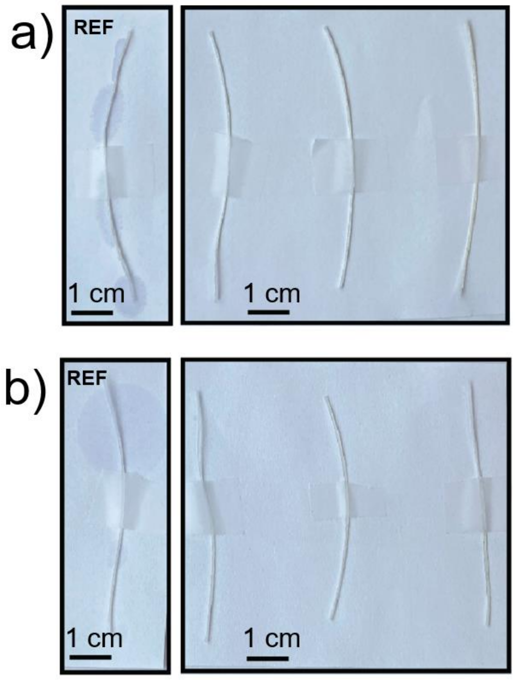

Figure S3. HF-PCM infill leakage test: a) HF-PEG leakage test (90 °C, 2h) the reference material (left) is an unsealed conduit. b) HF-PA leakage test (90 °C, 2h) the reference material (left) is an unsealed conduit.

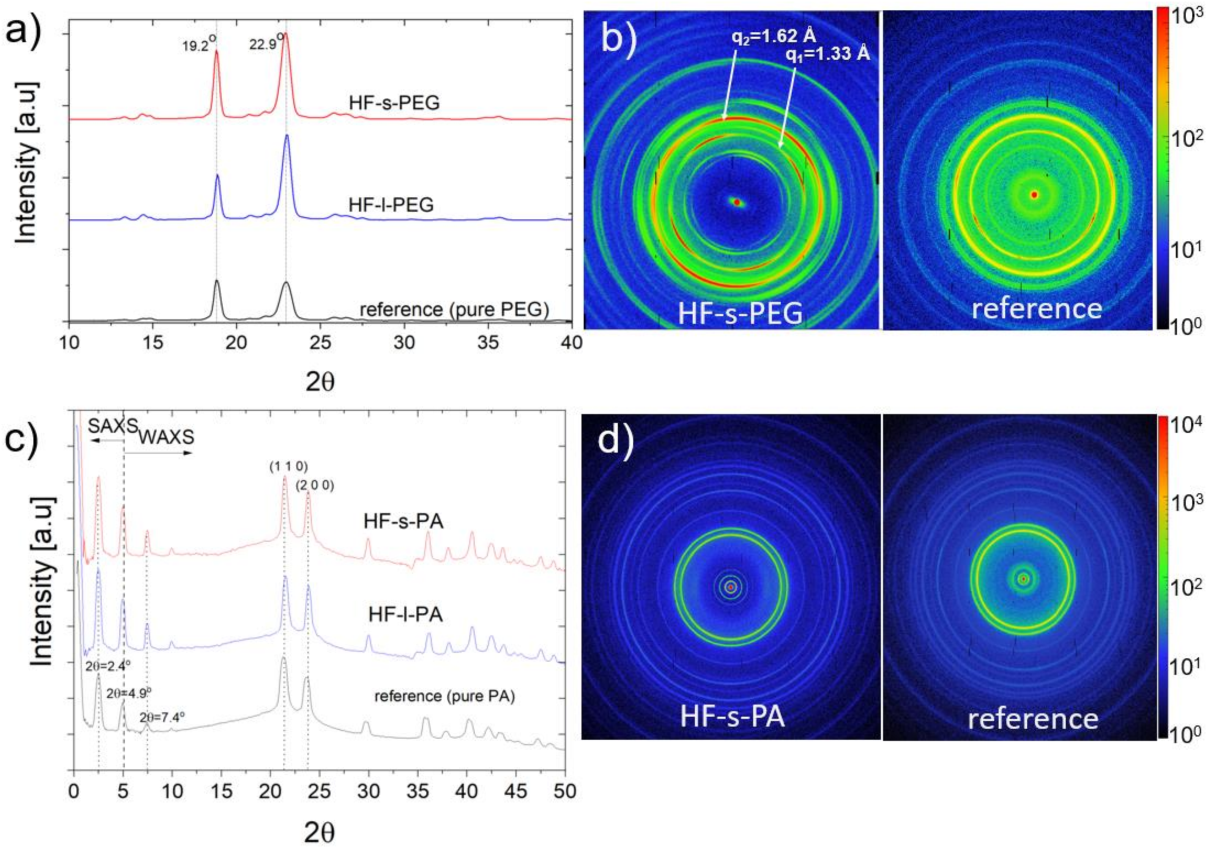

Figure S4. HF-PCM crystal structure: a) HF-PEG X-ray diffraction peaks (after removing the cellulose signal contribution). b) HF-s-PEG azimuthal peaks intensities. c) HF-PA SAXS/WAXS diffraction peaks (cellulose signal removed). d) HF-s-PA azimuthal peaks intensities.

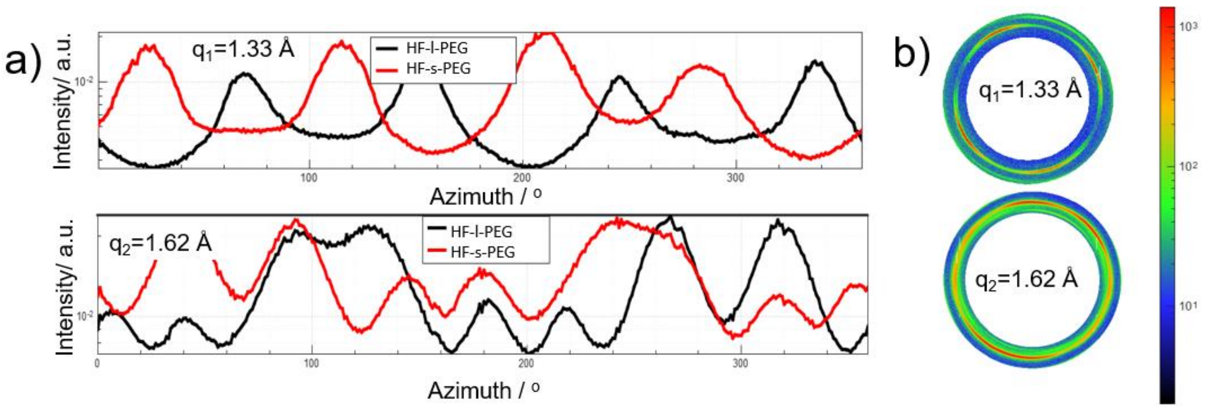

Figure S5. HF-PEG azimuthal intensities profiles: a) intensities distribution for peak q_1=1.33 Å and q_2=1.62 Å, respectively. b) azimuthal intensities integration for Herman's parameters computation.

PEG HF's exhibited two peaks at q_1=1.33 Å or (2θ=19.2 º), and q_2=1.62 Å or (2θ=22.9 º). The first peak at q_1, presented Herman's parameters of P_2= 0.18 (0.01), P_2 = -0.02 (0.01), and P_2= 0.01(0.001) for the small, large and reference materials respectively. The second peak (q_2) did not exhibit preferential orientation in any case; all Herman's parameter orientation values were around zero in this case, with P_2= 0.03(0.01), P_2 = -0.05(0.02), and P_2= 0.01(0.001) for the small, large and reference materials respectively.

Table S1. HF's X-rays diffraction peaks and crystal structure

**Sample**	**Peak position (deg)**							
	**(1-10)**	**(110)**	**(102)**	**(200)**	**(004)**	**Am.**	**CI [%]**	**τ [Å]**
Small	14.9	16.3	20.5	22.4	34.6	18.1	68	36
Medium	14.8	16.5	20.5	22.4	34.6	18.6	80	36
Large	14.9	16.5	20.5	22.4	34.6	18.1	78	35
					Average		75	35
					Standard deviation		6	1

Table S2. HF's structure parameters

**Size**	**Diameter φ [µm]**	**Wall thickness [µm]**	**BET surface area [$m^2.g^{-1}$]**	**BET pore size [nm]**	**Herman´s orientation P.***
Small	384 ≥ φ ≥ 265	28 (7)	6.2 (2.1)	3.1 (1)	0.18 (0.01)
Medium	503 ≥ φ ≥ 385	17 (5)	3.1 (0.4)	3.5 (0.3)	0.16 (0.00)
Large	630 ≥ φ ≥504	14 (3)	2.1 (0.2)	3.4 (0.4)	0.13 (0.03)

*The main peak with miller index (2 0 0) was selected to compute the orientation analysis distribution using **equation 3**

Table S3. HFs Herman´s orientation parameters

Parameter	Sample	Small	Medium	Large
Herman's Parameter	1	0.19	0.16	0.16
	2	0.19	0.16	0.10
	3	0.16	0.15	0.15
Average		0,18	0.16	0.13
Standard deviation		0,01	0.00	0.03
Mean orientation angle	1	180	176	179
	2	180	177	172
	3	178	178	179
Average		179	177	177
Standard deviation		1	1	4
Degree of orientation	1	23	19	18
	2	22	19	11
	3	19	18	17
Average		22	19	15
Standard deviation		2	1	3

Table S4. HF's mechanical parameters

Sample	Modulus [GPa]	Strength [MPa]	Strain %	Toughness [MJ·m^{-3}]	Density [g·cm^{-3}]	Porosity* [%]
Small	9 (2)	66 (19)	3 (1)	2.1 (0.5)	1.4 (0.1)	12 (9)
Medium	6 (2)	45 (4)	2 (1)	0.7 (0.1)	0.9 (0.1)	42 (7)
Large	5 (1)	49 (9)	3 (1)	1.3 (0.4)	0.8 (0.1)	45 (3)
Small-wet	0.9 (0.1)	15 (8)	6.5 (3)	0.8 (0.1)		
Medium-wet	0.9 (0.4)	9 (5)	6.5 (4)	0.4 (0.2)		
Large-wet	0.47 (0.08)	5 (3)	5 (3)	0.1(0.1)		

*The apparent porosity was computed with reference to the theoretical density of a cellulose fiber (1.55 g.cm^{-3})[1].

Table S5. HF-s-PCM's mechanical properties in the dry state

Sample	Modulus [GPa]	Strength [MPa]	Strain %	Toughness [MJ·m^{-3}]
HM-s	9 (2)	66 (19)	3 (1)	2.1 (0.5)
HM-s-PEG	3 (1)	39 (17)	4 (1)	1.1 (0.5)
HM-s-PA	4 (2)	51 (9)	5 (2)	1.6 (0.5)

Table S6. HF-s-PCM's mechanical properties in wet state

Sample	Modulus [MPa]	Strength [MPa]	Strain %	Toughness [kJ·m^{-3}]
HM-s	910 (140)	15 (8)	7 (3)	880 (170)
HM-s-PEG	36 (10)	3 (1)	9 (2)	82 (70)
HM-s-PA	30 (19)	2 (1)	8 (2)	73 (33)

HF-PCM's thermal properties

The following definition for enthalpy in terms of pressure and temperature *H(T,P)* summarizes the energy mechanisms that need to be considered for PCM's.

$$dH = \left(\frac{\partial H}{\partial T}\right)_P . \mathrm{dT} + \left(\frac{\partial H}{\partial P}\right)_T . \mathrm{dP} \ (S1)$$

where *H* is the enthalpy for an open system under a reversible process[2]; by definition, the first term in **equation S1** accounts for all the sensible heat. The second term encloses the latent heat during the phase transition. During a process where the PCM undergoes a thermodynamic process from an initial temperature (T_i) and final temperature (T_f) with a transition from the phase (α) to (β), the integration of **equation S1** results in the following expression. [2,3]

$$\Delta H_{total} = \int_{Ti}^{T\alpha} Cp_{\alpha} dT + \int_{T\beta}^{Tf} Cp_{\beta} dT + \Delta H^{\alpha\beta} \ (S2)$$

Where Cp_{α} and Cp_{β} are the specific heat capacities at constant pressure for the phase (α) and (β), respectively, and $\Delta H^{\alpha\beta}$ is the latent heat during the phase transition.

Table S7. HF-s-PCM's thermal properties at different cycles

**Cycle**	**Tm [˚C]**	**ΔH_m [J·g^{-1}]**	**Tc [˚C]**	**ΔH_c [J·g^{-1}]**	**Cp,s [J·g^{-1}·K^{-1}]**	**Cp,l [J·g^{-1}·K^{-1}]**	**PCM loading**
	HM-s-PA						
3	58.2	156.6	43.6	-155.5	2.47	2.59	74 %
50	57.7	152.5	43.9	-151.3			
100	57.9	152.9	43.8	-151.2			
	HM-s-PEG						
3	58.8	137.2	32.2	-130.7	1.32	1.87	75 %
50	59.1	137.7	31.5	-131.1			
100	62.7	137	32.4	-131.1			

REFERENCES

(1) Dufresne, A. Nanocellulose: A New Ageless Bionanomaterial. *Mater. Today* **2013**, *16* (6), 220–227. https://doi.org/10.1016/j.mattod.2013.06.004.

(2) Sandler, S. I. *Chemical, Biochemical, and Engineering Thermodynamics.*, 4th ed.; Willey-VCH GmbH and Co, 2006, 960.

(3) Kumar, N.; Gupta, S. K.; Sharma, V. K. Application of Phase Change Material for Thermal Energy Storage: An Overview of Recent Advances. *Mater. Today Proc.* **2021**, *44*, 368–375. https://doi.org/10.1016/j.matpr.2020.09.745.
